# A New Synthetic Methodology in the Preparation of Bimetallic Chalcogenide Clusters via Cluster-to-Cluster Transformations

**DOI:** 10.3390/molecules26175391

**Published:** 2021-09-05

**Authors:** Yu-Jie Zhong, Jian-Hong Liao, Tzu-Hao Chiu, Yuh-Sheng Wen, C. W. Liu

**Affiliations:** 1 Department of Chemistry, National Dong Hwa University, Hualien 974301, Taiwan; 410412037@gms.ndhu.edu.tw (Y.-J.Z.); d9912005@gms.ndhu.edu.tw (J.-H.L.); 410412055@gms.ndhu.edu.tw (T.-H.C.); 2 Institute of Chemistry, Academia Sinica, Taipei 11528, Taiwan; wenys@gate.sinica.edu.tw

**Keywords:** chalcogenide, hydride, silver, copper, inverse coordination

## Abstract

A decanuclear silver chalcogenide cluster, [Ag_10_(Se){Se_2_P(O*^i^*Pr)_2_}_8_] (**2**) was isolated from a hydride-encapsulated silver diisopropyl diselenophosphates, [Ag_7_(H){Se_2_P(O*^i^*Pr)_2_}_6_], under thermal condition. The time-dependent NMR spectroscopy showed that **2** was generated at the first three hours and the hydrido silver cluster was completely consumed after thirty-six hours. This method illustrated as cluster-to-cluster transformations can be applied to prepare selenide-centered decanuclear bimetallic clusters, [Cu_x_Ag_10-x_(Se){Se_2_P(O*^i^*Pr)_2_}_8_] (x = 0–7, **3**), via heating [Cu_x_Ag_7−x_(H){Se_2_P(O*^i^*Pr)_2_}_6_] (x = 1–6) at 60 °C. Compositions of **3** were accurately confirmed by the ESI mass spectrometry. While the crystal **2** revealed two un-identical [Ag_10_(Se){Se_2_P(O*^i^*Pr)_2_}_8_] structures in the asymmetric unit, a co-crystal of [Cu_3_Ag_7_(Se){Se_2_P(O*^i^*Pr)_2_}_8_]_0.6_[Cu_4_Ag_6_(Se){Se_2_P(O*^i^*Pr)_2_}_8_]_0.4_ ([**3a**]_0.6_[**3b**]_0.4_) was eventually characterized by single-crystal X-ray diffraction. Even though compositions of **2**, [**3a**]_0.6_[**3b**]_0.4_ and the previous published [Ag_10_(Se){Se_2_P(OEt)_2_}_8_] (**1**) are quite similar (10 metals, 1 Se^2−^, 8 ligands), their metal core arrangements are completely different. These results show that different synthetic methods by using different starting reagents can affect the structure of the resulting products, leading to polymorphism.

## 1. Introduction

In pursuit of metal chalcogenide clusters, Group 11 elements (Cu, Ag, Au) are frequently employed in the synthesis of novel clusters [1,2,3,4]. Silver chalcogenide clusters have rich structural varieties which can be synthesized by many different approaches [5,6,7,8,9,10,11]. The primary strategy is the reaction of different Ag(I) precursors with highly reactive silylated chalcogen reagents E(SiMe_3_)_2_ (E = S, Se, Te) [5], which can afford S^2−^/Se^2−^/Te^2−^ in the construction of high-nuclearity silver chalcogenide clusters stabilized by phosphine, chalcogenolate, halide, carboxylate, or alkynyl ligands [6,7,8,9,10,11]. Under this guideline, Fenske and his co-workers have structurally characterized many remarkable silver chalcogenide clusters [6,7]. For example, [Ag_114_Se_34_(Se*^n^*Bu)_46_(P*^t^*Bu_3_)_14_], which contains a distorted cubic structure, was synthesized involving the reaction of C_11_H_23_CO_2_Ag, *^n^*BuSeSiMe_3_ and P*^t^*Bu_3_ at low temperature (<−20 °C) [6]. Different diphosphine ligands (bis(diphenylphosphinol)propane, dppp) used in the previous reaction at −30 °C produce [Ag_172_Se_40_(Se*^n^*Bu)_92_(dppp)_4_], which not only increases the cluster nuclearity but also keeps similar cross sections of Ag_2_Se as that found in Ag_114_Se_34_ [6]. Another mega cluster synthesized by the reaction of CF_3_CO_2_Ag, dppm, PhS(SiMe_3_) and S(SiMe_3_)_2_ at −40 °C yielded [Ag_70_S_20_(PhS)_28_(dppm)_10_](CF_3_CO_2_)_2_ [7]. Compared with those giant silver chalcogenide clusters which are the kinetic products formed in different reaction temperatures, smaller silver chalcogenide clusters encapsulated with a single S^2−^/Se^2−^ are rarely reported [12,13,14,15,16,17,18,19,20,21,22]. The as-synthesized chalcogenide anion can easily insert in the metal clusters to achieve a high coordination number, i.e., μ_8_-Se in [Ag_8_(Se){Se_2_P(O*^i^*Pr)_2_}_6_] [15,16], μ_9_-Se in [Ag_11_(Se)(X)_3_{Se_2_P(OR)_2_}_6_] (X = I, Br; R = Et, *^i^*Pr, ^2^Bu) [17,18], μ_10_-Se in [Ag_10_(Se){Se_2_P(OEt)_2_}_8_] [19] and μ_12_-S in [Cu_12_(S){S_2_CNR_2_}_6_{C≡CR’}_4_] [22]. These hyper-coordinated anions, which become the coordination center surrounded by an array of metal atoms, are examples of inverse coordination, an emerging concept coined by Ionel Haiduic [23,24]. Nevertheless, efficient controls on both the amount of chalcogenide generated in situ and the size of clusters remain challenging. Herein, we report a new synthetic pathway leading to the formation of M_10_(Se)L_8_ (L = diisopropyl diselenophosphate, dsep) via a cluster-to-cluster transformation. In addition, intriguing structural isomers identified in the M_10_(Se) core are also presented.

## 2. Results and Discussion

### 2.1. Synthetic Strategy

**Scheme 1:**
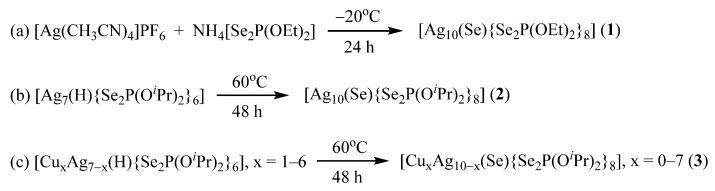
(**a**) The synthetic pathway of previous published [Ag_10_(Se){Se_2_P(OEt)_2_}_8_] (**1**). (**b**) A new pathway for the synthesis of [Ag_10_(Se){Se_2_P(O*^i^*Pr)_2_}_8_] (**2**) and (**c**) [Cu_x_Ag_10−x_(Se){Se_2_P(O*^i^*Pr)_2_}_8_] (x = 0–7, **3**).

In our previous study, a decanuclear silver cluster, [Ag_10_(Se){Se_2_P(OEt)_2_}]_8_ (**1**), was isolated from the reaction of [Ag(CH_3_CN)_4_]PF_6_ and NH_4_[Se_2_P(OEt)_2_] in a 1:1 molar ratio at −20 °C for 24 h (Scheme 1a) [19]. The encapsulated selenide anion was generated from the slow decomposition of dsep ligands. Herein we introduced a new strategy, which is inspired by the thermal-induced self-redox reaction of [Ag_7_(H){S_2_P(O*^i^*Pr)_2_}_8_] leading to the generation of a two-electron silver superatom, [Ag_10_{S_2_P(O*^i^*Pr)_2_}_8_] [25]. In this work, the Se-analogue of [Ag_7_(H){S_2_P(O*^i^*Pr)_2_}_8_] [26] as precursors under heating (Scheme 1b) can yield [Ag_10_(Se){Se_2_P(OEt)_2_}]_8_ (**2**). The composition of **2** (10 Ag^+^ + 1 Se^2−^ + 8 dsep ligands) has been characterized by X-ray diffraction, which is the same as **1** but with a completely different solid-state structure. At 60 °C the cleavage of P-Se bond of dsep ligands occurs to generate Se, which can be reduced to Se^2−^ by the interstitial hydride in [Ag_7_(H){Se_2_P(O*^i^*Pr)_2_}_8_]. Unlike the reactions used silylated chalcogen reagents to generate considerable amount of chalcogenide anions, this new method can control the Se^2−^ ratio in a relatively small range so that smaller size of metal chalcogenide clusters can be generated.

Following the same strategy, a diselenophosphate-stabilized bimetallic Cu/Ag hydride, [Cu_x_Ag_7-x_(H){Se_2_P(O*^i^*Pr)_2_}_6_] (x = 1–6) [26] was used instead of [Ag_7_(H){Se_2_P(O*^i^*Pr)_2_}_8_] (Scheme 1c), to form a selenide-centered decanuclear bimetallic cluster, [Cu_x_Ag_10−x_(Se){Se_2_P(O*^i^*Pr)_2_}_8_] (x = 0–7) (**3**). This methodology provides a facile route to produce anion-encapsulated heterometallic clusters via a cluster-to-cluster transformation. However, this method cannot predict the exact position where the heterometals will possibly occupy. Nevertheless, it opens up many possibilities to generate structural isomers. Compound **3**, which the entire metal core is completely different from that of **1** and **2**, has been structurally characterized by XRD. The structural analysis will be discussed in the following section.

### 2.2. Structural Analyses

**Figure 1:**
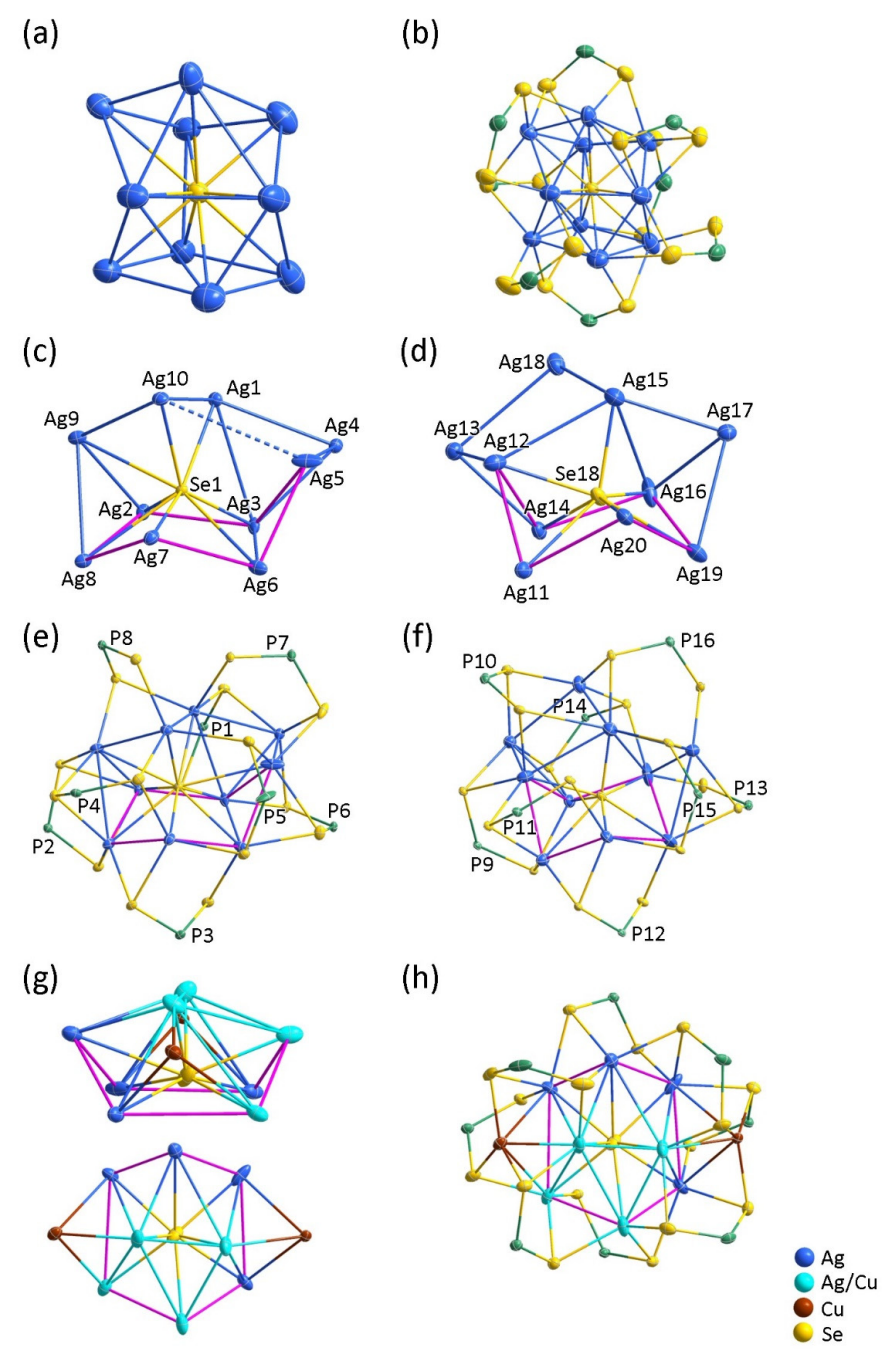
(**a**) The Ag_10_(Se) core and (**b**) the total structure of previously published [Ag_10_(Se){Se_2_P(OEt)_2_}_8_] (**1**). (**c**) The Ag_10_(Se) cores of cluster A and (**d**) cluster B in newly characterized [Ag_10_(Se){Se_2_P(O*^i^*Pr)_2_}_8_] (**2**). (**e**) The total structure of cluster A and (**f**) cluster B in **2**. (**g**) The Cu_x_Ag_10-x_(Se) (x = 3.4) core and (**h**) the total structure of [**3a**]_0.6_[**3b**]_0.4_. (Thermal ellipsoid plots were drawn at 30% probability with isopropoxy groups omitted for clarity).

All three M_10_(Se)L_8_ clusters (**1**–**3**) are composed of ten metal atoms, one selenide anion and eight dsep ligands. Although three structures have similar compositions, their geometries are strikingly different. In the previously published structure **1**, the selenide anion is encapsulated in a distorted, *cis*-bicapped trapezoidal-prismatic Ag_10_ framework (Figure 1a), which is surrounded by eight dsep ligands (Figure 1b) [19]. Short contacts are observed between the encapsulated Se^2−^ (Se_encap_) and ten peripheral silver atoms where Se_encap_-Ag distances are in the range of 2.6312(19)–3.187(2) Å (avg. 2.939(2) Å).

In structure **2**, the metal array is quite different from **1**. There are two clusters in the asymmetric unit and their structures are slightly different. Considering the two Ag_10_(Se) cores in crystal **2** as cluster A (Figure 1c) and cluster B (Figure 1d), both clusters have six out of ten Ag atoms (Ag_chair_) arranged in a chair-like metallo-ring conformation with one Se^2−^ anion sitting above its center. The rest of four Ag atoms are located above the (Ag_chair_)_6_Se unit to constitute the whole Ag_10_(Se) framework. Since the positions of the ten silver atoms in clusters A and B are slightly different, two Ag_10_(Se) cores are pseudo-mirror images. This also affects the distances of Ag-Se_encap_, resulting in different coordination modes of the central Se atom in clusters A and B. Se1 has eight short contacts to the adjacent Ag atoms ranging from 2.5113(13) to 3.0596(12) Å (avg. 2.7586(13) Å) in cluster A (Figure 1c); seven short contacts between Se2 and Ag in cluster B (Figure 1d), which range from 2.5279(12) to 3.0496(12) Å (avg. 2.8271(12) Å). Both Se_encap_-Ag distances in **2** are slightly shorter than those in **1**, indicating stronger interactions between Ag and Se_encap_. The average Ag-Ag distances in clusters A (3.0780(12) Å) and B (3.0786(13) Å) are not much different. The eight dsep ligands in clusters A (Figure 1e) and B (Figure 1f) have roughly the same locations around the Ag_10_Se core, i.e., P7 and P16, P8 and P10, etc. However, their coordination patterns at relatively similar position are not the same due to the different metal sites in each Ag_10_(Se) core. For example, the dsep ligand on P8 is trimetallic (Ag1, Ag10, Ag9) triconnectivity, but P10 is tetrametallic (Ag15, Ag18, Ag13, Ag12) tetraconnectivity. Nine of the ten Ag atoms are three-coordinated and one Ag atom (Ag3) are two-coordinated to Se on the dsep ligands in cluster A; eight of the ten Ag atoms are trigonal- and two Ag atoms (Ag14 and Ag16) are digonal-coordinated in cluster B. There are 29 and 28 connectivities between dsep ligands and Ag_10_(Se) core in clusters A and B, respectively, which Se-Ag distances are in the range of 2.5368(13)–3.0478(14) Å (avg. 2.6672(13) Å) in cluster A and 2.5496(13)–3.0811(12) Å (avg. 2.693(2) Å) in cluster B. Cluster **1** displays 30 Se-Ag connectivities with slightly longer distances ranging from 2.577(3) to 3.127(3) Å (avg. 2.685(3) Å). These metric data strongly suggest that **1** and clusters A, B of **2** exhibit not only minute different dsep-silver bonding patterns on the outer shell but also intrinsically different Ag_10_(Se) cores, leading to unusual polymorphism.

The molecular formula in structure **3**, [Cu_x_Ag_10−x_(Se){Se_2_P(O*^i^*Pr)_2_}_8_] (x = 3.4), was determined by X-ray diffraction. Non-integer Cu atoms were satisfactorily refined, which corresponded to the co-crystallization of 60% of [Cu_3_Ag_7_(Se){Se_2_P(O*^i^*Pr)_2_}_8_] (**3a**) and 40% of [Cu_4_Ag_6_(Se){Se_2_P(O*^i^*Pr)_2_}_8_] (**3b**). Hence, the crystal structure can be best represented as [Cu_3_Ag_7_(Se){Se_2_P(O*^i^*Pr)_2_}_8_]_0.6_[Cu_4_Ag_6_(Se){Se_2_P(O*^i^*Pr)_2_}_8_]_0.4_ ([**3a**]_0.6_[**3b**]_0.4_). It is worthwhile to mention that the phenomenon of co-crystallization is frequently observed in heterometallic clusters [26,27,28,29,30,31] especially for two heterometals occupying the same site. The copper atoms in M_10_(Se) are distributed in six positions, where two Cu are fully occupied at two brown ellipsoids (Figure 1g) and the rest Cu atoms are randomly disordered at four cyan ellipsoids (see Figure S1). Compared with **2**, the bottom six metal atoms display a M_6_ boat conformation with a Se^2−^ anion at its center. The other two metals are on the top of the M_6_(Se) motif and two Cu atoms on each side. It shows that the Se^2−^ anion binds to the six M_boat_ atoms and two metals on the top with Se_encap_-M distances of 2.425(2)–3.127(2) Å (avg. 2.778(2) Å). Distances of Se17-Cu5B(Ag5) and Se17-Cu6B(Ag6), 2.842(2) and 2.908(2) Å, are relatively long, exhibiting weaker interactions while Cu occupation. Distances between the fully occupied Cu and the Se_encap_ atom are approximately 4.7 Å which are much longer than the distances (3.6–4.2 Å) of three un-bonded Ag atoms to Se_encap_ in **2**. Noticed that the M_10_(Se) framework in [**3a**]_0.6_[**3b**]_0.4_ (Figure 1h, bottom) is partially similar to the

Ag_10_(Se) framework in **1**. If the two Cu atoms in [**3a**]_0.6_[**3b**]_0.4_ and the two Ag atoms from the back in **1** are removed, respectively, the leftover M_8_(Se) motifs are in similar atomic arrangement (Figure S2). Nevertheless, the arrangement of eight dsep ligands in [**3a**]_0.6_[**3b**]_0.4_ has no similarity to the eight ligands in **1**. The top two metals are two-coordinated and the rest eight metals are three-coordinated to Se atoms of dsep ligands (Figure 1f). Se-M distances range in 2.351(2)–2.8797(19) Å (avg. 2.593(2) Å) are shorter than those in **1** and **2**, which can be attributed to the smaller covalent radii of the partially doped Cu atoms. Interestingly, structures **2** and [**3a**]_0.6_[**3b**]_0.4_ display a unique Ag_6_ chair and a M_6_ boat in the metal array, respectively. The averaged adjacent, Ag_chair_-Ag_chair_ and M_boat_-M_boat_ distances are 3.0622(14) Å (cluster A in **2**), 3.0987(12) Å (cluster B in **2**) and 3.076(2) Å (in [**3a**]_0.6_[**3b**]_0.4_), indicating strong metallophilic interactions. Selected bond lengths are summarized in Table 1.

### 2.3. ESI Mass Spectroscopy

**Figure 2:**
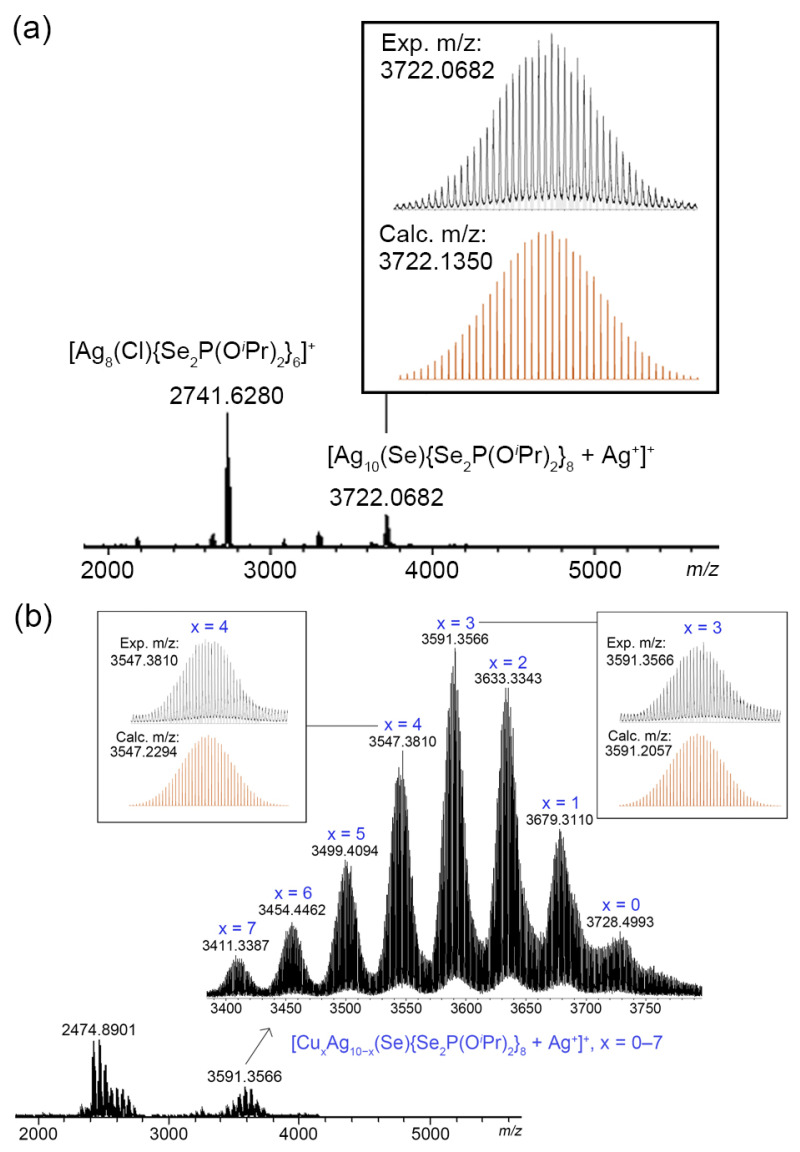
(**a**) The positive-ion ESI mass spectrum of **2**. The inset is the experimental (black) and simulated (orange) isotopic distribution pattern of [**2** + Ag^+^]^+^. (**b**) The positive-ion ESI mass spectrum of **3**. The inset is the experimental (black) and simulated (orange) isotopic distribution pattern of [Cu_3_Ag_7_(Se){Se_2_P(O*^i^*Pr)_2_}_8_ + Ag^+^]^+^ and [Cu_4_Ag_6_(Se){Se_2_P(O*^i^*Pr)_2_}_8_ + Ag^+^]^+^.

The positive-ion ESI-MS spectrum of **2** (Figure 2a) shows an adduct ion peak at *m/z* 3722.0682, corresponding to the whole cluster with an additional silver ion, [**2** + Ag^+^]^+^ (calc. *m/z*: 3722.1350). Another intense peak at *m/z* 2741.6280 can be formulated as [Ag_8_(Cl){Se_2_P(O*^i^*Pr)_2_}_6_]^+^ (calc. *m/z* 2741.6542). Presumably, the chloride is from CH_2_Cl_2_ used to dissolve samples for the ionization. The spectrum of **3** (Figure 2b) showed a wide distribution of [Cu_x_Ag_10-x_(Se){Se_2_P(O*^i^*Pr)_2_}_8_ + Ag^+^]^+^ (x = 0–7) with the most intense peak of x = 3. The inset spectra depict the experimental isotopic distribution patterns of [Cu_3_Ag_7_(Se){Se_2_P(O*^i^*Pr)_2_}_8_ + Ag^+^]^+^ (exp. 3591.3566, calc. 3591.2057) and [Cu_4_Ag_6_(Se){Se_2_P(O*^i^*Pr)_2_}_8_ + Ag^+^]^+^ (exp. 3547.3810, calc. 3547.2294), which are well-matched with the calculated ones and structures of both species have been identified by the single-crystal X-ray diffraction with the composition of [Cu_3_Ag_7_(Se){Se_2_P(O*^i^*Pr)_2_}_8_]_0.6_[Cu_4_Ag_6_(Se){Se_2_P(O*^i^*Pr)_2_}_8_]_0.4_ ([**3a**]_0.6_[**3b**]_0.4_). Although the spectrum was measured by using single crystals, we still observed the ion peak distributions (x = 0–7) depicted in the mass spectrum. The results can be attributed to the metal exchange in the gas phase. Another intense band around *m/z* 2474 can be assigned to [Cu_y_Ag_8-y_(Cl){Se_2_P(O*^i^*Pr)_2_}_6_]^+^ (y = 0–8) with the most intense peak (y = 3) at *m/z* 2474.8901 (calc. *m/z*: 2474.7998). Presumably, the [M_8_(Cl)L_6_]^+^ species identified in the gas phase are the decomposition products of M_10_(Se)L_8_ in the presence of chloro-solvent. In fact, the observed ion peak distributions for bimetallic clusters are frequently reported in literature [25,26,27,28].

### 2.4. NMR Spectroscopy

**Figure 3:**
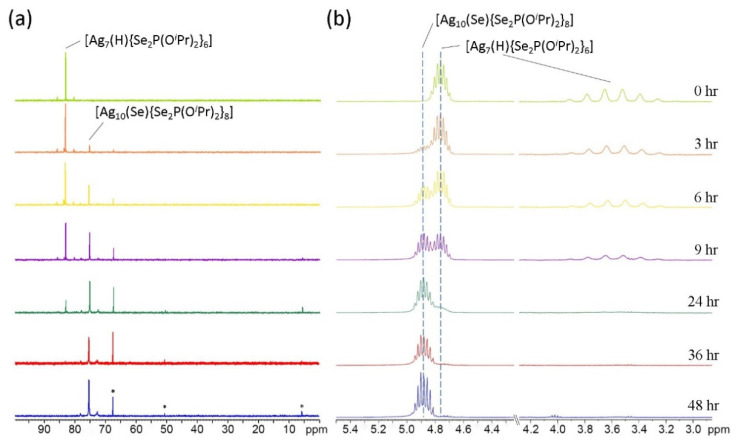
(**a**) Time-dependent ^31^P{^1^H} and (**b**) ^1^H NMR spectrum of the reaction acquired from 0 to 48 h (asterisks denote the fragments of dsep ligands after decomposition).

The fact that a single resonance centered at 75.3 ppm flanked by one set of satellite peaks (^1^*J*_PSe_ = 669 Hz) in the ^31^P{^1^H} NMR spectrum (Figure S3) coupled with one set of isopropoxy resonance (4.88 and 1.36 ppm) observed in the ^1^H NMR spectrum of **2** at ambient temperature (Figure S6) strongly suggests its non-rigid structure in solution, which could be due to the labile Ag-Se bonds. The satellite peaks are due to the ^31^P nuclei coupled with the ^77^Se nuclei (I = 1/2) in diselenophosphate compounds [32] in which the natural abundance of ^77^Se is only 7.56%. The cluster-to-cluster transformation process can be monitored by the time-dependent ^31^P{^1^H} and ^1^H NMR spectroscopy. In the reaction shown in Scheme 1b, compound **2** which ^31^P resonance at 75.3 ppm was generated at the first three hours (Figure 3a). The ^31^P resonance of [Ag_7_(H){Se_2_P(O*^i^*Pr)_2_}_6_] at 83.1 ppm gradually disappeared over time, then Ag_7_(H) was barely seen after 1.5 days. The consumption of Ag_7_(H) can also be followed by the time-dependent ^1^H spectra (Figure 3b). That is, the pseudo octet peak of central hydride originated from ^1^*J*(H, Ag) was gradually disappeared and the chemical shift of methine proton on isopropoxy groups displayed a down-field shift due to the different values of two species, Ag_7_H and Ag_10_Se. It can be assumed that the slow decomposition of Ag_7_(H) prevents Se^2−^ being released in large quantities at one time followed by the cluster-to-cluster transformation. The fact that the coordinated hydride as the reductant can be tracked by recognizing the in-situ generated H_2_, which resonates at 4.61 ppm (Figure S7). Fragment peaks from dsep ligand decomposition can also be observed in the ^31^P{^1^H} NMR spectrum. The chemical shifts at 67.4 (^1^*J*_PSe_ = 475, 848 Hz), 50.5 and 5.6 ppm belong to the resonance frequency of Se[P(Se)(O*^i^*Pr)_2_]_2_, [SeOP(O*^i^*Pr)_2_]^−^ [33] and phosphoric acid, respectively. It is noted that the diselenophosphates are thermally unstable where its sulfur analogue is not so sensitive to heat [34].

While the ^31^P chemical shifts of both **1** and **2** are almost identical, their ^77^Se{^1^H} NMR (Figure S4) spectra are different. A doublet at 103.3 ppm and a broad resonance at −1292.4 ppm (Figure S5) corresponding to the resonance frequency of the dsep ligands and the encapsulated Se^2−^, respectively, can be seen in the ^77^Se spectrum of **2**. The resonances are slightly different from that of **1** (108 ppm, dsep; −1395.4 ppm, Se_encap_) [19]. It could be due to the stronger interactions between Se_encap_ and Ag atoms in **2**, resulting in the downfield shift of the Se_encap_ resonance. The ^31^P{^1^H} NMR spectrum of **3** shows multiple resonances overlapping together at around 73.9–75.2 ppm (Figure S8), indicating the multiple coordination environment of the dsep ligands. It is primarily arisen from the randomly doped Cu atoms on multiple positions in structure **3**. Unfortunately, no satisfied signals can be detected in the ^77^Se NMR spectrum of **3** at ambient temperature.

### 2.5. Photophysical Properties

**Figure 4:**
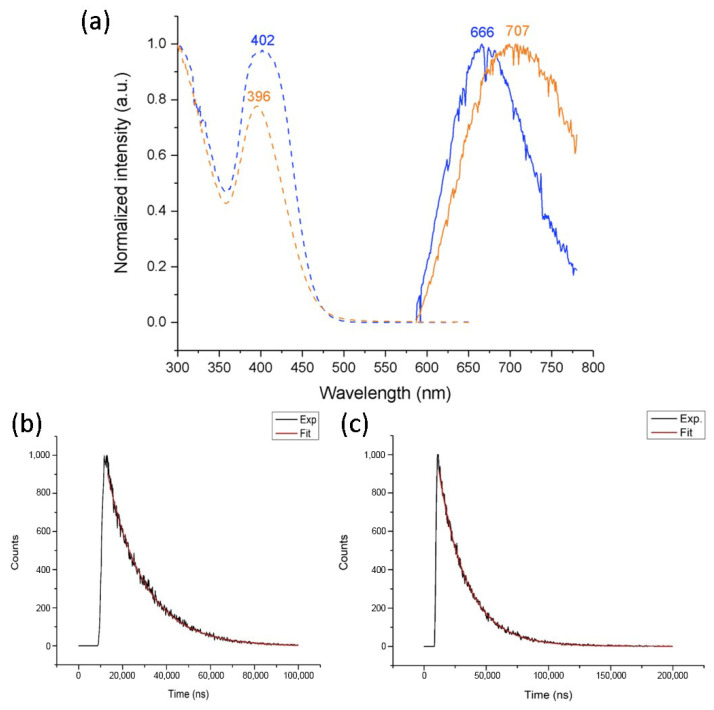
(**a**) The absorption spectrum of **2** (blue dashed line) and **3** (orange dashed line). The emission spectrum of **2** (blue solid line) and **3** (orange solid line) dissolved in 2-MeTHF at 77K. (**b**) The photoluminescence decay curves of **2** (λ_ex_ = 401 nm) and (**c**) **3** (λ_ex_ = 396 nm) in 2-MeTHF glass recorded at 77 K.

The absorption spectrum of **2** exhibits a single low-energy LMCT band at 402 nm, which is very close to 396 nm of **3** (Figure 4a). The doped Cu atoms found in the bimetallic decanuclear cluster seem not to significantly perturb the characteristic absorption band. It is assumed that both clusters **2** and **3** might have similar structures as that observed in cluster **1** in solution leading to similar electronic transition energy even though their solid-state structures are different (vide supra). Both compounds are not emissive at ambient temperature but show orange emission at 77 K. Figure 4a depicts the emission spectra of **2** and **3** in 2-MeTHF glass, while their photophysical data are briefly summarized in Table 2. Both emission profiles are structureless and the photoluminescence of **2** centered at 666 nm is slightly blue-shifted, compared to **3** centered at 707 nm. The photoluminescence decay curves of **2** (Figure 4b) and **3** (Figure 4c) can be well-fitted to a single exponential decay function (red curve). The lifetimes of **2** (λ_ex_ = 401 nm) and **3** (λ_ex_ = 396 nm), 16 μs and 22 μs, together with large Stokes shift (~10,000 cm^−1^), reflect their triplet excited state nature, a spin-forbidden phosphorescence.

## 3. Materials and Methods

### 3.1. General Remarks

All chemicals were purchased from commercial sources and used as received. Solvents were purified following standard protocols [35]. All reactions were performed in oven-dried Schlenk glassware using standard inert atmosphere techniques. All reactions were carried out under N_2_ atmosphere by using standard Schlenk techniques. [Ag_7_(H){Se_2_P(O*^i^*Pr)_2_}_6_] and [Cu_x_Ag_7-x_(H){Se_2_P(O*^i^*Pr)_2_}_6_] (x = 1–6) were prepared by the procedure reported earlier in literature [26]. ^1^H, ^31^P and ^77^Se NMR spectra were recorded on a Bruker Avance DPX-300 BBO probe spectrometer (Bruker BioSpin, MA, USA), operating at 300.13 MHz for ^1^H, 121.49 MHz for ^31^P and 57.239 MHz for ^77^Se, respectively. The chemical shift (δ) and coupling constant (*J*) are reported in ppm and Hz, respectively. ESI mass spectrum recorded on a Fison Quattro Bio-Q (Fisons Instruments, VG Biotech, Glasgow, UK). UV-visible absorption spectra were measured on a PerkinElmer Lambda 750 PerkinElmer, Inc., MA, USA) spectrophotometer using quartz cells with path length of 1 cm. Luminescence spectra and lifetime were recorded on an Edinburgh FLS920 fluorescence spectrometer (Edinburgh Instruments Ltd., Livingston, UK). The elemental analysis (C, H and N content) of the new compounds was determined by Elementar UNICUBE elemental analyzer (Elementar Analysensysteme GmbH, Langenselbold, Germany).

### 3.2. Synthesis and Characterization of Compounds **2**–**3**

#### 3.2.1. Synthesis and Characterization of [Ag_10_(Se){Se_2_P(O^i^Pr)_2_}_8_] (**2**)

[Ag_7_(H){Se_2_P(O*^i^*Pr)_2_}_6_] (0.0604 g, 0.0232 mmol) was dissolved in chloroform (30 mL). The temperature was elevated to 60 °C and kept stirring for 48 h. The solution was dried under vacuum to get a yellow solid. The compound can be purified by thin-layer chromatography (TLC) with a mixed solvent of dichloromethane and *n*-hexane in equal proportions (50:50, *v*/*v*). Yield: 0.0187 g (31.8%, based on Ag).

^31^P{^1^H} NMR (121.49 MHz, *d*-chloroform, δ, ppm): 75.3 (s, ^1^*J*_PSe_ = 669 Hz). ^77^Se NMR (57.24 MHz, *d*-chloroform, δ, ppm): 103.3 (d, ^1^*J*_PSe_ = 669 Hz, PSe_2_), −1292.4 (br, Ag_10_Se). ^1^H NMR (300.13 MHz, *d*-chloroform, δ, ppm): 4.88 (m, 16H, CH), 1.36 (d, 96H, CH_3_, ^3^*J*_HH_ = 6 Hz). Anal. calc. for C_48_H_112_Ag_10_O_16_P_8_Se_17_·2(CHCl_3_): C, 15.59; H, 2.98; Found: C, 15.67; H, 2.90%. ESI-MS (*m/z*): exp. 3722.0682 (calc. for [**2** + Ag^+^]^+^: 3722.1657).

#### 3.2.2. Synthesis and Characterization of [Cu_x_Ag_10-x_(Se){Se_2_P(O^i^Pr)_2_}_8_], x = 0–7 (**3**)

[Cu_x_Ag_7−x_(H){Se_2_P(O*^i^*Pr)_2_}_6_] (x = 1–6) was prepared by the slightly modified procedures reported earlier in literature [26]. ([Cu_7_(H){Se_2_P(O*^i^*Pr)_2_}_6_] and [Ag_7_(H){Se_2_P(O*^i^*Pr)_2_}_6_] with molar ratio 1:1). Followed the same synthetic procedure of **2**, [Cu_x_Ag_7−x_(H){Se_2_P(O*^i^*Pr)_2_}_6_] (0.050 g) was used as precursor instead of [Ag_7_(H){Se_2_P(O*^i^*Pr)_2_}_6_]. It was dissolved in chloroform (30 mL). The temperature was elevated to 60 °C and kept stirring for 48 h. The solution was dried under vacuum to get a yellow solid. The compound can be purified by thin-layer chromatography (TLC) with a mixed solvent of dichloromethane and *n*-hexane in equal proportions (50:50, *v*/*v*). Yield: 0.017 g (based on single crystals).

^31^P{^1^H} NMR (121.49 MHz, *d*-chloroform, δ, ppm): 75.2 (s, ^1^*J*_PSe_ = 667 Hz), 75.1 (s, ^1^*J*_PSe_ = 669 Hz), 74.9 (s, ^1^*J*_PSe_ = 669 Hz), 74.7 (s, ^1^*J*_PSe_ = 668 Hz), 74.5 (s, ^1^*J*_PSe_ = 666 Hz). No satisfied signals can be identified in ^77^Se NMR spectrum at room temperature. ^1^H NMR (400.13 MHz, *d*-chloroform, δ, ppm): 4.88 (m, 16H, CH), 1.36 (d, 96H, CH_3_, ^3^*J*_HH_ = 6 Hz). ESI-MS (*m/z*) of [Cu_x_Ag_10-x_(Se){Se_2_P(O^i^Pr)_2_}_8_ + Ag^+^]^+^: exp. 3411.3387 (calc. for x = 7: 3411.3034), exp. 3454.4462 (calc. for x = 6: 3454.2803), exp. 3499.4094 (calc. for x = 5: 3499.2556), exp. 3547.3810 (calc. for x = 4: 3547.2294), exp. 3591.3566 (calc. for x = 3: 3591.2057), exp. 3633.3343 (calc. for x = 2: 3633.1827), exp. 3679.3110 (calc. for x = 1: 3679.1578), exp. 3728.4993 (calc. for x = 0: 3728.1321).

### 3.3. X-ray Crystallography

Single crystals suitable for X-ray diffraction analysis of **2** and [**3a**]_0.6_[**3b**]_0.4_ were obtained by slow evaporation of acetone solution at ambient temperature within a week. Single crystals were mounted on the tip of glass fiber coated in paratone oil and then transferred into the cold N_2_ gas stream. Data were collected on a Bruker APEX II CCD diffractometer (Bruker AXS Inc., WI, USA) using graphite monochromated Mo K*α* radiation (*λ* = 0.71073 Å) at 100K. Absorption corrections for area detector were performed with SADABS [36] and the integration of raw data frame was performed with SAINT [37]. The structure was solved by direct methods and refined by least-squares against *F*^2^ using the SHELXL-2018/3 package [38], incorporated in SHELXTL/PC V6.14 [39]. All non-hydrogen atoms were refined anisotropically. The detailed refinements of occupancy ratio on each atomic site are listed in Figure S1. Selected X-ray crystallographic data are listed in Table 3.

## 4. Conclusions

Two decanuclear clusters, [Ag_10_(Se){Se_2_P(O*^i^*Pr)_2_}_8_] (**2**) and [Cu_x_Ag_10-x_(Se){Se_2_P(O*^i^*Pr)_2_}_8_] (x = 0–7) (**3**), have been successfully synthesized via the cluster-to-cluster transformation from the starting reagents, [M_7_(H)(dsep)_6_], under thermal condition. The newly developed synthetic methodology provides a facile way to produce both homometallic and heterometallic chalcogenide clusters. Surprisingly, although the composition of [Ag_10_(Se){Se_2_P(O*^i^*Pr)_2_}_8_] (**2**) is almost identical to the previously published [Ag_10_(Se){Se_2_P(OEt)_2_}_8_] (**1**), the whole molecular shape in **2** is completely different from **1**. This can be attributed to the use of different synthetic methods resulting in structural diversity. We also identify polymorphism on **2**, where two pseudo symmetric [Ag_10_(Se){Se_2_P(O*^i^*Pr)_2_}_8_] clusters are co-existed in the same asymmetric unit. Furthermore, a bimetallic chalcogenide cluster **3** can be elegantly generated by using a bimetallic hydride as the precursor. It is anticipated that a variety of metal chalcogenide clusters can be synthesized via a cluster-to-cluster transformation in the near future.

## Figures and Tables

**Table 1:** Selected bond lengths of **1, 2** and [**3a**]_0.6_[**3b**]_0.4_.

Comp.	1	2		[3a]_0.6_[3b]_0.4_
Ag-Ag/M-M (Å)	2.957(3)–3.378(2), avg. 3.083(3)	Clust. A	2.8786(12)–3.3004(11), avg. 3.0786(13)	2.8625(15)–2.7856(18), avg. 3.036(2)
		Clust. B	2.9186(11)–3.3321(11), avg. 3.0780(12)	
Se_encap_-Ag/Se_encap_-M (Å)	2.6312(19)–3.187(3), avg. 2.939(2)	Clust. A	2.5279(12)–3.0496(12), avg. 2.8271(12)	2.425(2)–3.127(2), avg. 2.778(2)
		Clust. B	2.5113(13)–3.0596(12), avg. 2.7586(13)	
Se-Ag/Se-M (Å)	2.557(3)–3.127(3), avg. 2.685(3)	Clust. A	2.5496(13)–3.0811(12), avg. 2.693(2)	2.351(2)–2.8797(19), acg. 2.593 (2)
		Clust. B	2.5368(13)–3.0478(14), avg. 2.6672(13)	

**Table 2:** Photophysical data of **2** and **3**.

Comp.	λ_abs_/nm (ε/cm ^−1^ M^−1^)	λ_ex_/nm (at 77K)	λ_em_/nm (at 77K)	Stoke Shift/cm^−1^	Lifetime/ μs (at 77K)
**2**	402 (14,600)	384	666	9900	16
**3**	396 (11,500)	387	707	11,100	22

**Table 3:** Selected X-ray crystallographic data of **2** and [**3a**]_0.6_[**3b**]_0.4_.

Compound	2	[3a]_0.6_[3b]_0.4_
Chemical formula	C_48_H_112_Ag_10_O_16_P_8_Se_17_	C_48_H_112_Ag_6.6_Cu_3.4_O_16_P_8_Se_17_
Formula weight	3614.15	3463.43
Crystal System	Triclinic	Triclinic
Space group	*P*(-)1	*P*(-)1
a, Å	14.7908(9)	14.8584(4)
b, Å	24.8040(16)	14.8871(5)
c, Å	27.5596(17)	25.6577(7)
α, deg.	83.563(2)	100.1735(13)
β, deg.	85.402(2)	96.5137(13)
γ, deg.	82.033(2)	115.4953(15)
V, Å^3^	9928.6(11)	4926.2(3)
Z	4	2
Temperature, K	100(2)	100(2)
ρ_calcd_, g/cm^3^	2.418	2.335
μ, mm^−1^	8.335	8.458
θ_max_, deg.	25.000	24.999
Completeness, %	97.5	100
Reflection collected/unique	53,934/34,122 [R_int_ = 0.0383]	167,862/17,346 [R_int_ = 0.2349]
Restraints/parameters	876/1888	382/937
^a^ R1, ^b^ wR2 [I > 2σ(I)]	0.0490, 0.1010	0.0555, 0.1029
^a^ R1, ^b^ wR2 (all data)	0.0714, 0.1082	0.1139, 0.1290
GOF	1.006	1.023
Largest diff. peak and hole, e/Å^3^	2.269 and −2.512	2.287 and −2.157

^a^ R1 = Σ||F_o_| − |Fc||/Σ|F_o_|. ^b^ wR2 = {Σ[w(F_o_^2^ − F_c_^2^)^2^]/Σ[w(F_o_^2^)^2^]}^1/2^.

## Data Availability

The data presented in this study are available on request from the corresponding authors. CCDC 2098961 (**2**) and 2098962 ([**3a**]_0.6_[**3b**]_0.4_) contains the supplementary crystallographic data for compounds **2** and [**3a**]_0.6_[**3b**]_0.4_ in this article. These data can be obtained free of charge from The Cam-bridge Crystallographic Data Centre via www.ccdc.cam.ac.uk/data_request/cif.

## Supplementary Materials

The following are available online. X-ray refinement, NMR spectra and photophysical data. Figure S1: The M_10_(Se) framework of [**3a**]_0.6_[**3b**]_0.4_, (thermal ellipsoid plots were drawn at the 30 % probability level), with the refined occupancy ratios on each position. Figure S2: (a) The schematic representation of Ag_8_(Se) skeleton in **1** and (b) M_8_(Se) skeleton in [**3a**]_0.6_[**3b**]_0.4_. Figure S3: ^31^P{^1^H} NMR spectrum of **2**. Figure S4: ^77^Se NMR spectrum of **2**. Figure S5: ^77^Se NMR spectrum of **2**. Figure S6: ^1^H NMR spectrum of **2**. Figure S7: The magnified time-dependent ^1^H NMR spectrum during the reaction of **2**. Figure S8: ^31^P{^1^H} NMR spectrum of **3**. Figure S9: ^1^H NMR spectrum of **3**.

## Sample Availability

Samples of the compounds are available from the authors.
