# Supplementary material for: A New Synthetic Methodology in the Preparation of Bimetallic Chalcogenide Clusters via Cluster-to-Cluster Transformations
Source: Molecules. 2021 Sep 5;26(17):5391. doi: 10.3390/molecules26175391 (PMC8433971; doi:10.3390/molecules26175391)
Supplement: Supplementary file 1 [file molecules-26-05391-s001.zip › molecules-1355945-supplementary.pdf]

Supplementary Information

A new synthetic methodology in the preparation of bimetallic chalcogenide clusters via cluster-to-cluster transformations

Yu-Jie Zhong [1], Jian-Hong Liao [1], Tzu-Hao Chiu [1], Yuh-Sheng Wen [2], and C. W. Liu [1,*]

[1] Department of Chemistry, National Dong Hwa University, Hualien 974301, Taiwan (Republic of China)

[2] Institute of Chemistry, Academia Sinica, Taipei 11528, Taiwan (Republic of China)

***** Correspondence: chenwei@mail.ndhu.edu.tw; Tel.: +886-3-890-3607;

http://faculty.ndhu.edu.tw/~cwl/index.htm

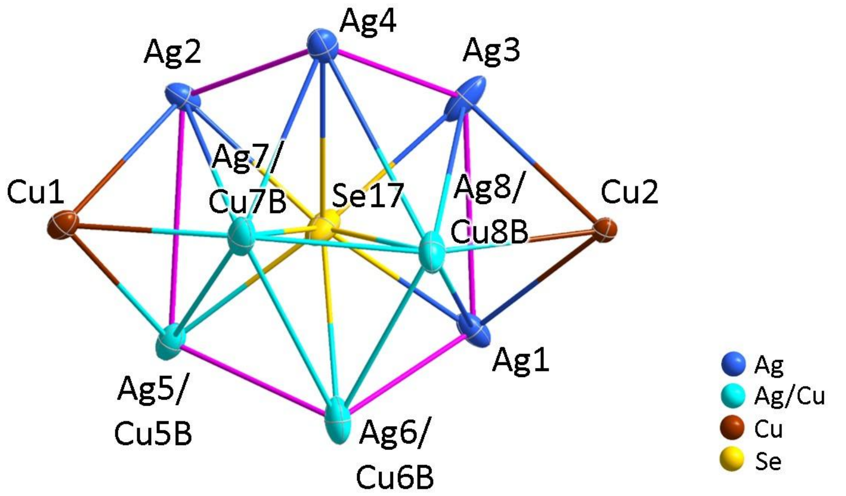

Atomic site	Cu1	Cu2	Ag1	[a]Ag2/ Ag2B	Ag3	Ag4	Ag5/ Cu5B	Ag6/ Cu6B	Ag7/ Cu7B	Ag8/ Cu8B	Total occupancy
Cu occupancy	1	1	0	0	0	0	0.2	0.2	0.5	0.5	3.4
Ag occupancy	0	0	1	0.8/ 0.2	1	1	0.8	0.8	0.5	0.5	6.6
Total occupancy	1	1	1	1	1	1	1	1	1	1	10

[a] The Ag2 was disordered and split into two sites (Ag2 and Ag2B) with occupancy ratio 80:20. Herein, only the major distribution is discussed.

Figure S1. The M_{10}(Se) framework of [**3a**]$_{0.6}$[**3b**]$_{0.4}$, (thermal ellipsoid plots were drawn at the 30 % probability level), with the refined occupancy ratios on each position.

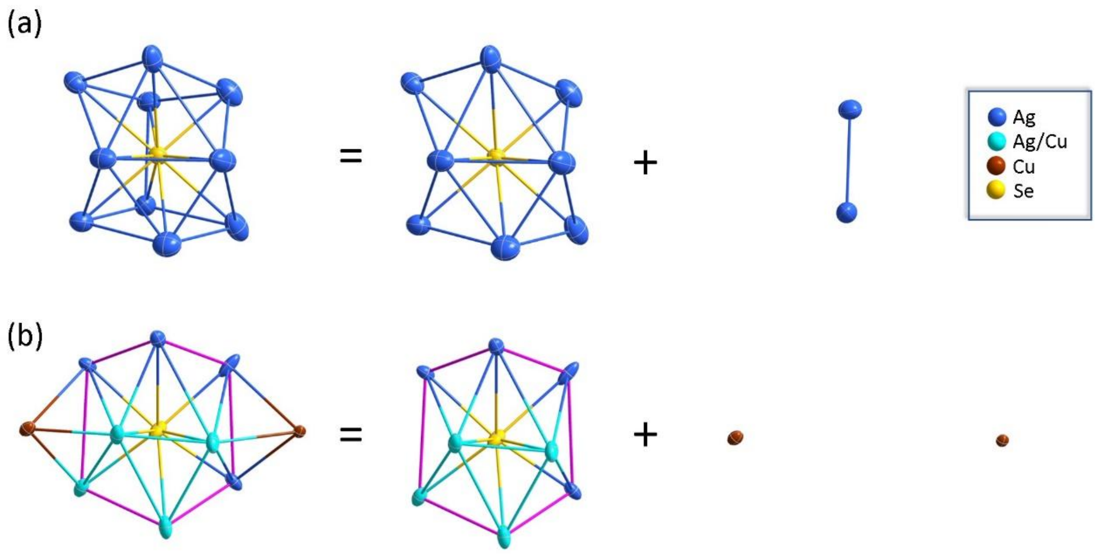

Figure S2. (a) The schematic representation of $Ag_8(Se)$ skeleton in **1** and (b) $M_8(Se)$ skeleton in [**3a**]$_{0.6}$[**3b**]$_{0.4}$.

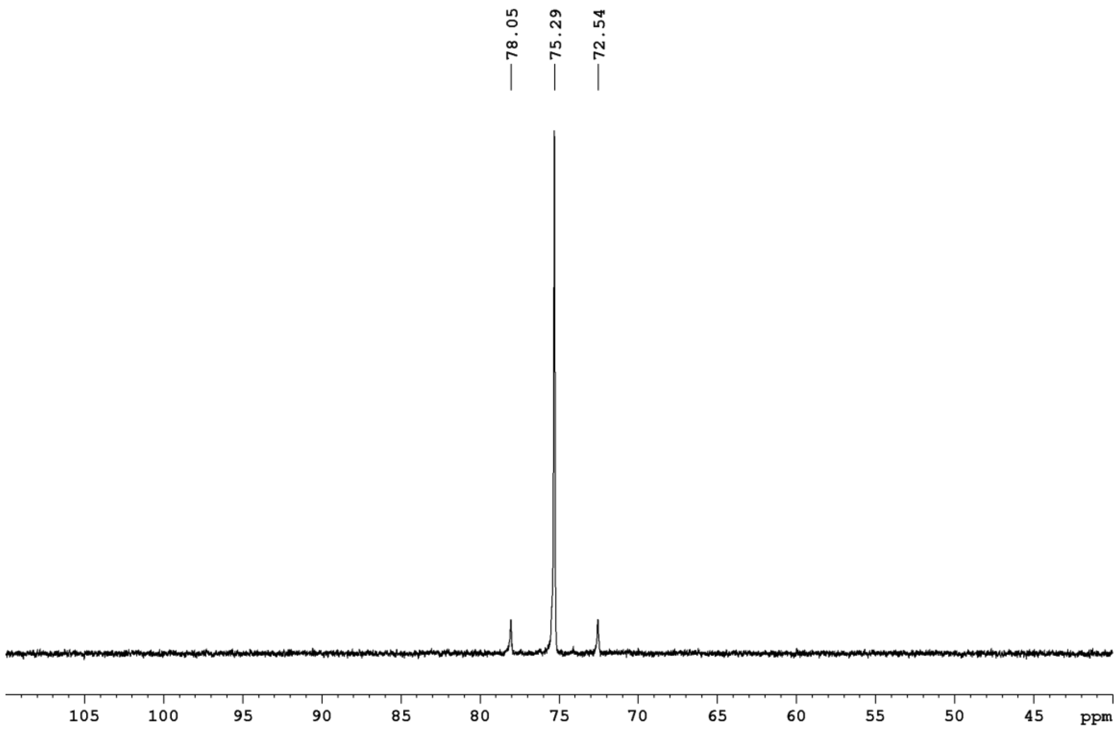

Figure S3. $^{31}P\{^{1}H\}$ NMR spectrum of **2**.

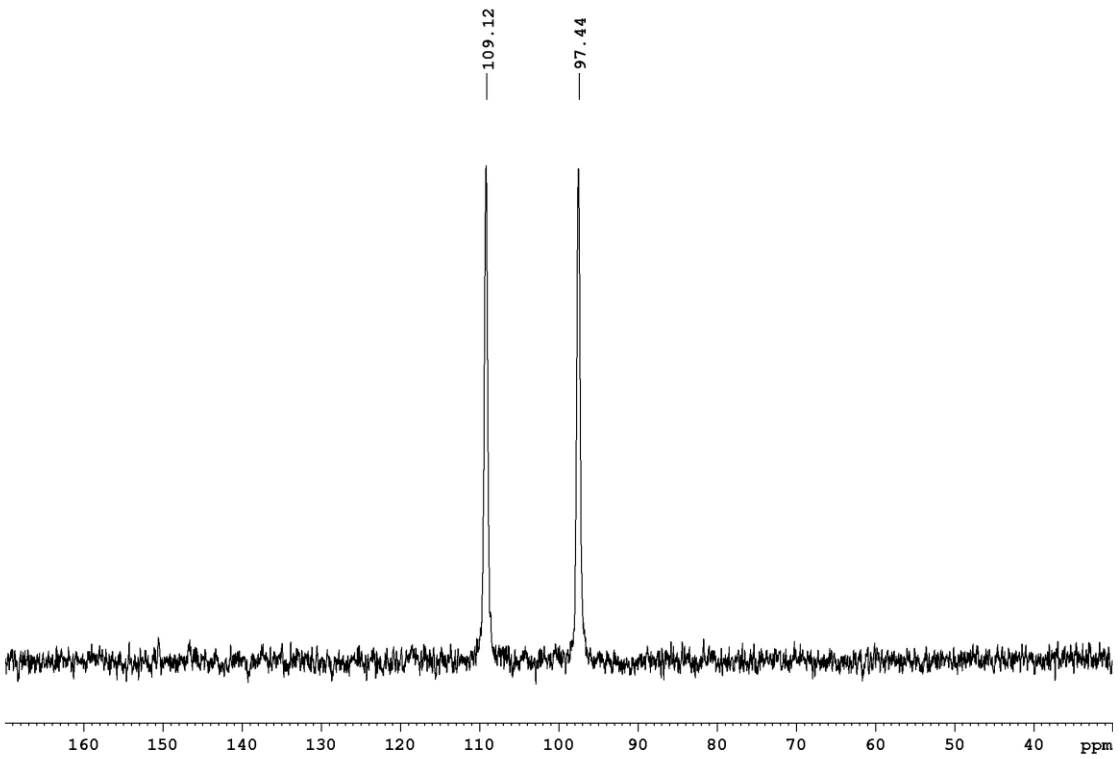

Figure S4. ^{77}Se NMR spectrum of **2**.

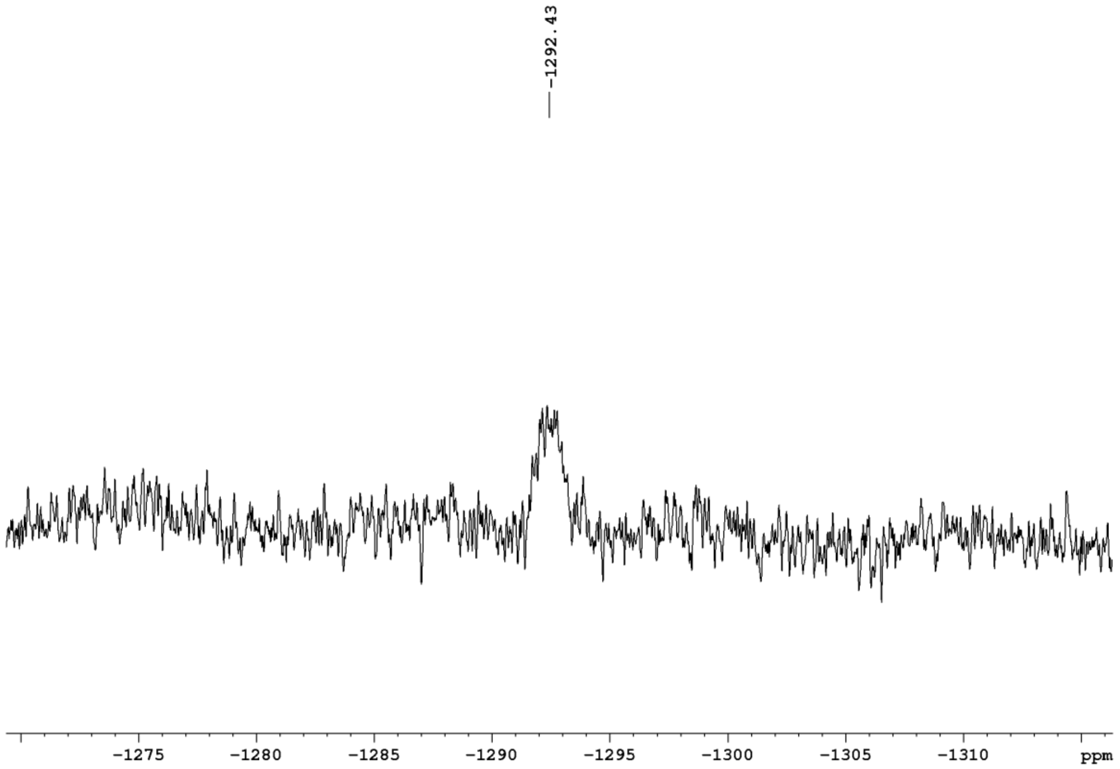

Figure S5. ^{77}Se NMR spectrum of **2**.

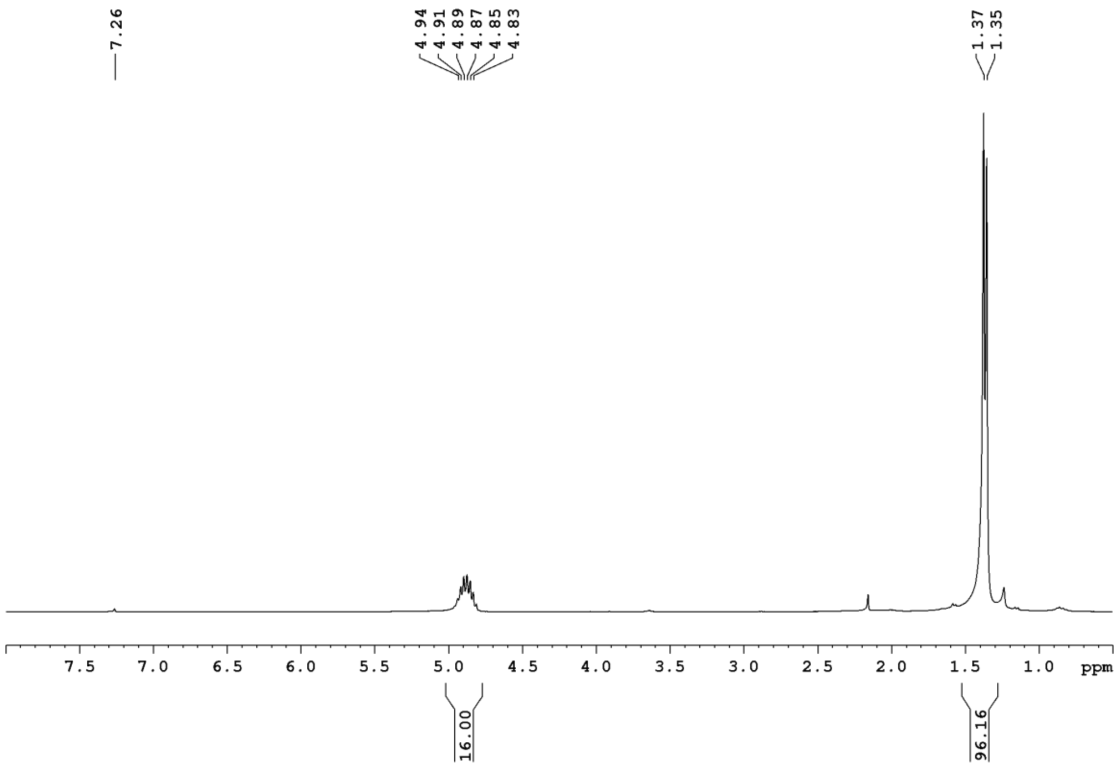

Figure S6. ^{1}H NMR spectrum of **2**.

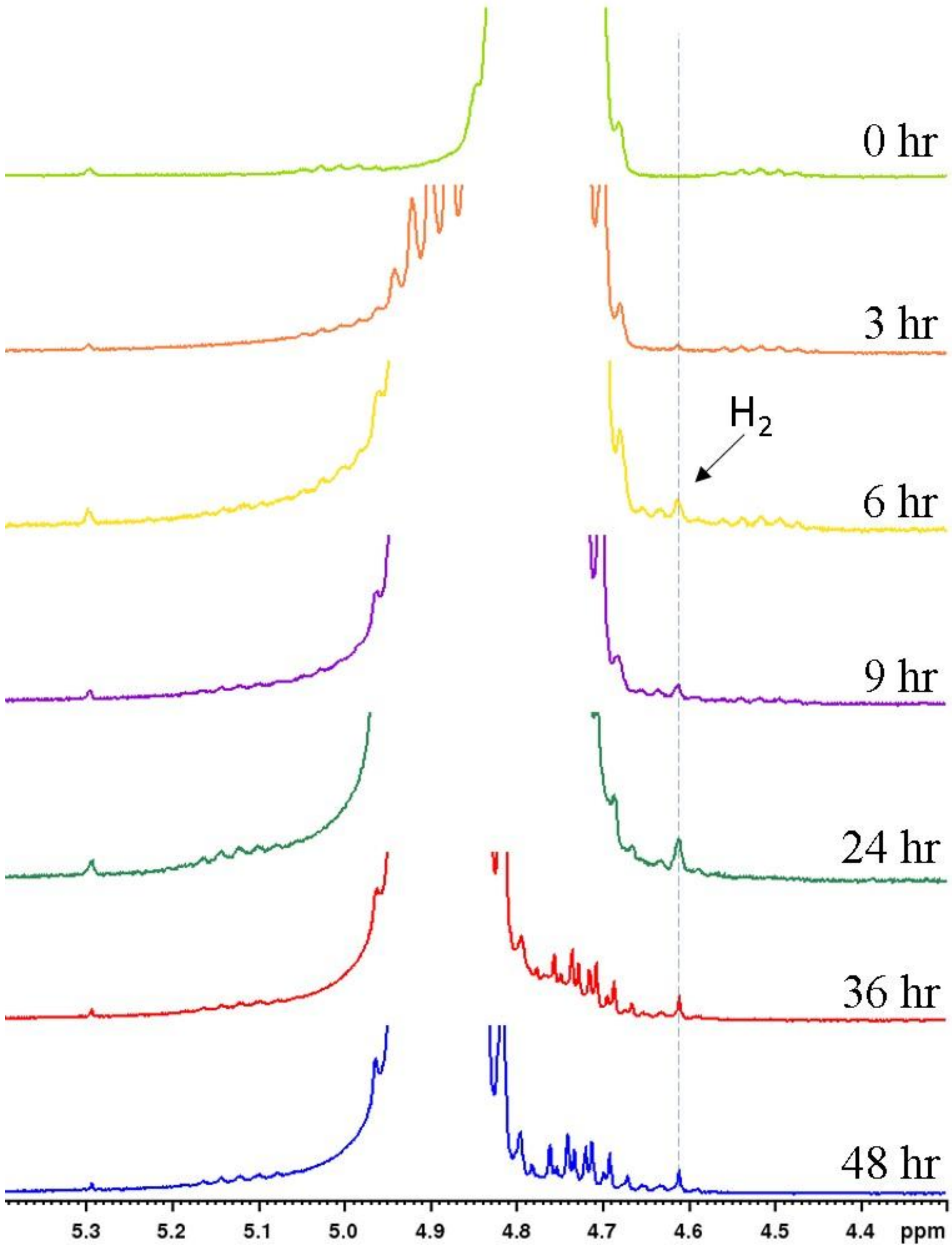

Figure S7. The magnified time-dependent ^{1}H NMR spectrum during the reaction of **2**.

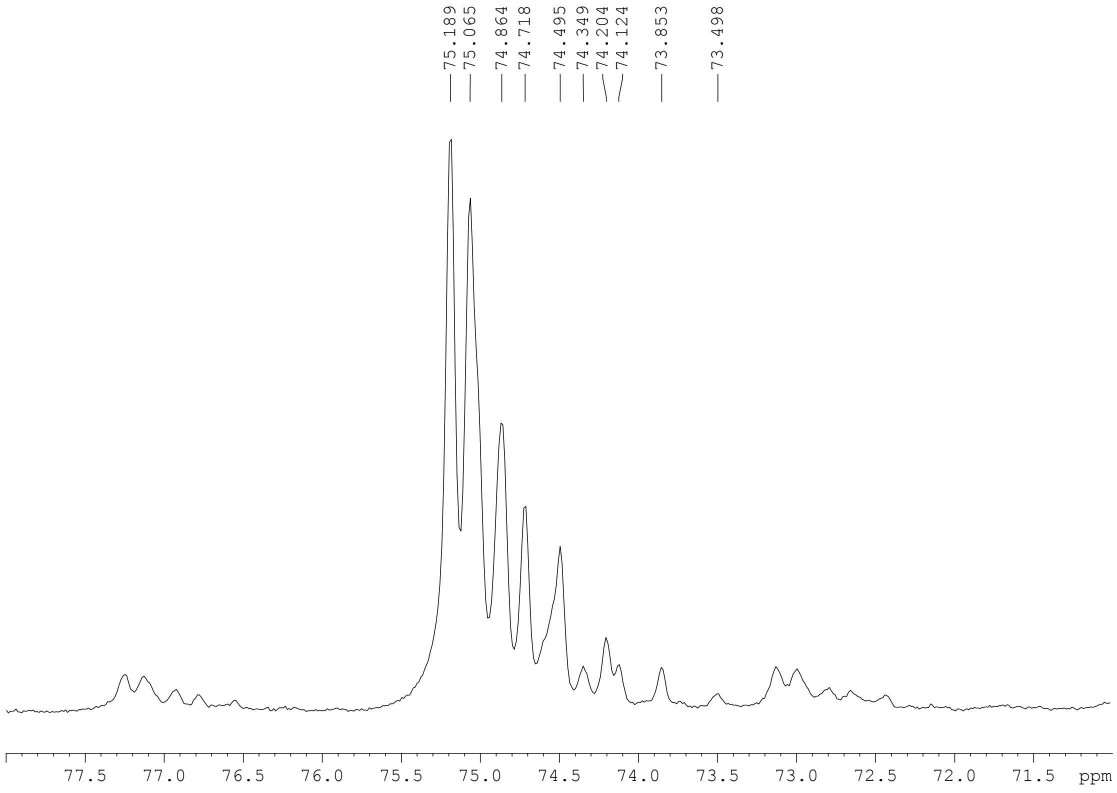

Figure S8. $^{31}P\{^{1}H\}$ NMR spectrum of **3**.

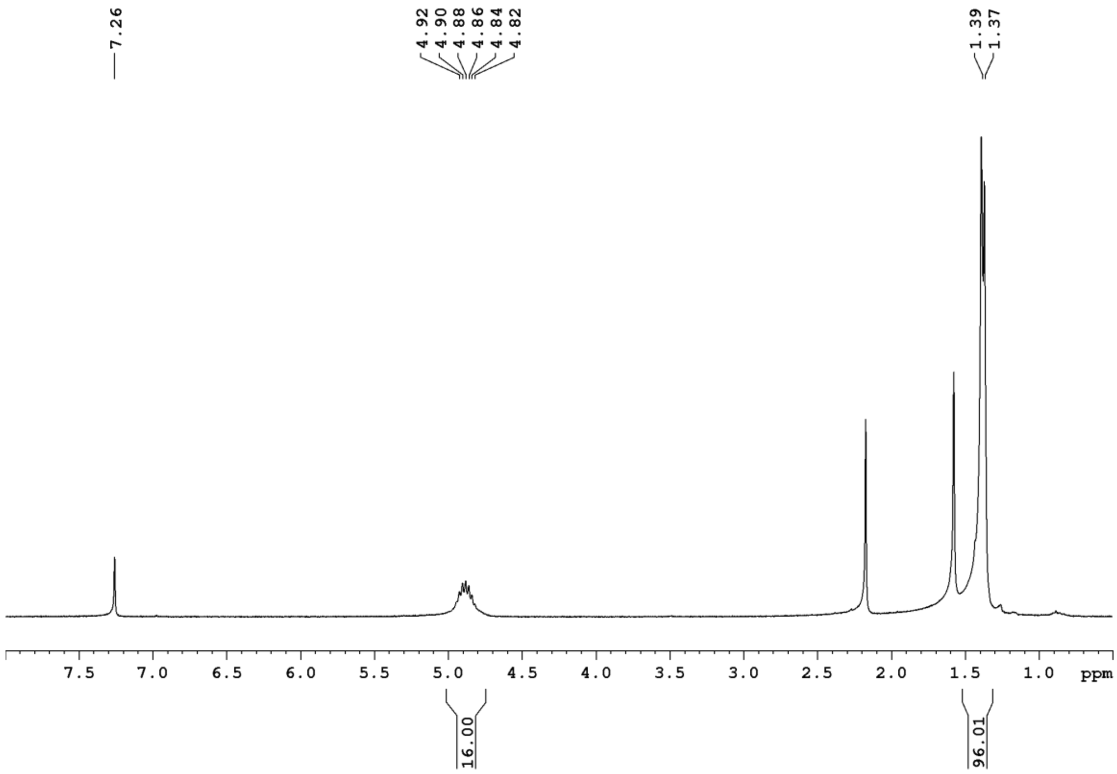

Figure S9. ^{1}H NMR spectrum of **3**.
